# Versatility of ARD1/NAA10-mediated protein lysine acetylation

**DOI:** 10.1038/s12276-018-0100-7

**Published:** 2018-07-27

**Authors:** Tam Thuy Lu Vo, Chul-Ho Jeong, Sooyeun Lee, Kyu-Won Kim, Eunyoung Ha, Ji Hae Seo

**Affiliations:** 1 College of Pharmacy, Keimyung University, Daegue, 42601 Republic of Korea; 2 College of Pharmacy and Research Institute of Pharmaceutical Sciences, Seoul National University, Seoul, 08826 Republic of Korea; 3 Department of Biochemistry, Keimyung University School of Medicine, Daegu, 42601 Republic of Korea

**Keywords:** Protein folding, Protein folding

## Abstract

Post-translational modifications (PTMs) are chemical alterations that occur in proteins that play critical roles in various cellular functions. Lysine acetylation is an important PTM in eukaryotes, and it is catalyzed by lysine acetyltransferases (KATs). KATs transfer acetyl-coenzyme A to the internal lysine residue of substrate proteins. Arrest defective 1 (ARD1) is a member of the KAT family. Since the identification of its KAT activity 15 years ago, many studies have revealed that diverse cellular proteins are acetylated by ARD1. ARD1-mediated lysine acetylation is a key switch that regulates the enzymatic activities and biological functions of proteins and influences cell biology from development to pathology. In this review, we summarize protein lysine acetylation mediated by ARD1 and describe the biological meanings of this modification.

## Protein modification: Effect on activity

Enzymes that modify proteins by adding an acetyl group have profound effects on metabolism and development, as well as disease. This process, known as acetylation, is carried out by KAT proteins, which are present throughout the body. Although acetyl groups are small, acetylation can change a protein’s electrical charge and shape, and even alter its function. Ji Hae Seo at Keimyung University School of Medicine in Daegu, South Korea, and co-workers reviewed the roles of KAT proteins in health and disease. They report that KAT proteins control gene expression, switch metabolic pathways on or off, and regulate development. Malfunction can lead to various disorders, including neurodegeneration and tumor growth. The researchers highlight several KAT proteins, in particular an enzyme that acetylates the amino acid lysine, that are promising targets for treatment of diseases, including cancer.

## Introduction

**Fig. 1:**
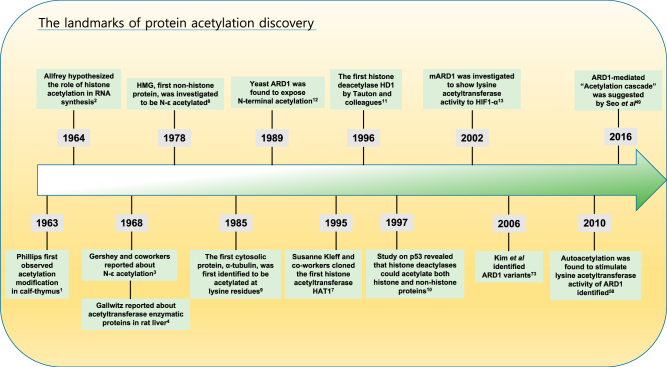
Timeline of breakthroughs that laid the foundation for the development of protein acetylation studies in developmental and pathological processes

Post-translational modifications (PTMs) are an issue because they govern many biological processes during the development and disease by modifying the end products of expression. Acetylation is a major PTM in cells, along with phosphorylation, methylation, and ubiquitination. The first acetylation modification was identified in histone by Phillips in 1963 when he fractionated calf thymus nuclei to analyze histone components^1^. In 1964, Allfrey hypothesized that the acetylation of histone plays a critical role in the regulation of RNA synthesis^2^. Subsequently, many studies on histone modification, particularly histone acetylation, were performed. In 1968, in addition to the observation of Phillips that histone acetylation mainly occurred at the N-terminus of the polypeptide chain, Gershey et al. reported that histone was modified after synthesis by the attachment of acetyl groups to the ε-amino nitrogen of the lysine residues in the polypeptide chain^3^. In the same year, Gallwitz reported the occurrence of proteins that exhibited acetyltransferase activity in rat liver nuclei^4^. In the following years, many attempts to purify and characterize histone acetyltransferases (HATs) have been recorded in different organisms^5,6^. In 1995, Kleff et al. identified and cloned the first HAT, HAT1^7^. A decade after the first description of histone acetylation, in 1978, the first non-histone protein, high-mobility group (HMG) was found to be acetylated at the ε-amino nitrogen of its lysine residues^8^. In 1985, cytosolic protein α-tubulin was first shown to be acetylated at a lysine residue^9^. However, it was not known that HATs could also acetylate non-histone proteins until the acetylation of p53 was identified in 1997^10^. Thereafter, the broader term lysine acetyltransferases (KATs) was used instead of HATs. Although the deacetylation of lysine residues was believed to oppose N-ε-acetylation, and together with lysine acetylation, modulate cellular functions, the mechanism underlying deacetylation was unclear until Tauton et al. cloned and characterized the first histone deacetylase, HD1, in 1996^11^. These groundbreaking findings have helped the study of protein acetylation for not only histone acetylation but also non-histone protein acetylation. Mullen et al. screened for yeast mutants with defective HAT activity and found that *nat1*, which is identical to *ard1*, was involved in N-terminal acetyltransferase (NAT) activity^12^. Thereafter, mouse ARD1 was reported to harbor KAT activity in addition to N-terminal acetyltransferase activity in a complex with NAT1^13^. Herein, we provide an overview of protein acetylation. In particular, we focus on discussing the contributions of ARD1 to protein lysine acetylation (Fig. 1).

## Protein acetylation and deacetylation

### Acetylation

Protein acetylation is a modification in which the acetyl group is transferred from the donor coenzyme A to either the Nα-termini of nascent polypeptide chains (termed N-terminal acetylation) or specific lysine residues on the polypeptide chains (termed lysine acetylation). N-terminal acetylation is a ubiquitous event in eukaryotes, as its rate of occurrence is more than 80% in humans and approximately 70% and 60% in *Drosophila* and yeast, respectively^14,15^. N-terminal acetylation has long been known to occur co-translationally and irreversibly. Nonetheless, previous studies have revealed that this modification also possibly occurs in a post-translational manner^16,17^.

In contrast to the irreversible reaction of N-terminal acetylation, post-translational lysine acetylation is catalyzed by the “writers” or KATs, which add acetyl groups to proteins, and is reversed by the “erasers” or lysine deacetylases (KDACs), which remove the acetyl groups from proteins^18^. Lysine acetylation is highly conserved across prokaryotes and eukaryotes^19^. The balance between lysine acetylation and deacetylation is tightly administered to ensure appropriate gene transcription, signaling machinery, and metabolism regulation^20^. Lysine acetylation neutralizes the positive charges that form on the amino group, and as a result, it has a significant impact on the electrostatic properties and conformation of the protein, thereby causing changes in the functions of proteins that regulate diverse biological events in the cytoplasm, nucleus, and mitochondria, such as the DNA-binding affinity of transcription factors, protein-protein interactions, and protein stability^18^. The acetylation of proteins at lysine residues is the signal to switch on or off signaling pathways or metabolic pathways, recognized by the “readers” that contain the recognizing domains, the so-called bromodomains^21^. Lysine acetylation also harmonically interplays with other modifications, such as phosphorylation, ubiquitination, and methylation, to regulate cellular events^22^. Therefore, malfunction of in vivo acetylation results in the improper regulation of biological processes, leading to disorders and diseases, namely, neurodegeneration, atherosclerosis, aneurysm, myocardial infarction, or tumorigenesis^19^.

Similar to the kinase proteins, KATs represent internal lysine acetylation and the acetylation of substrate proteins. Autoacetylation of KATs is essential for the acetyltransferase activity of KATs, as observed in the autoacetylation of ARD1, Tip60, MOF, p300, and p300/CBP-associated factor (PCAF). On the basis of sequence identity, KATs have been classified into three primary families, namely, the GNAT, MYST, and p300/CBP families (Table 1).

**Table 1:** Classification and properties of lysine acetyltransferases and histone deacetylases

*KATs*					
*Class*	*Representatives*	*Autoacetylation ability*	*Diversity*		
GNAT	GCN5	N/A	Prokaryotes and all eukaryotes		
	PCAF	Yes			
	HAT1	N/A			
	ARD1	Yes			
	Nut1, Elp3, Hpa2/Hpa2, MCM3AP, and Eco1	N/A			
MYST	HBO1 (MYST2 or KAT7)	N/A	Prokaryotes and all eukaryotes		
	MOF (MYST1 or KAT8)	Yes			
	MORF (MYST4 or KAT6B)	Yes			
	MOZ (MYST3 or KAT6)	N/A			
	TIP60 (KAT5)	Yes			
p300/CBP	p300/CBP	Yes	Metazoan specific		
	Rtt109	Yes	Yeast specific		
*HDACs*					
*Class*	*Members*	*Subcellular localization*	*Yeast homologous protein*	*Cofactor*	*Tissue expression*
I	HDAC1	Nucleus	Rpd3	Zn^2+^	Ubiquitous
	HDAC2	Nucleus			
	HDAC3	Nucleus/Cytoplasm			
	HDAC8	Nucleus			
IIA	HDAC4	Nucleus/Cytoplasm	Hda1	Zn^2+^	Specific
	HDAC5	Nucleus/Cytoplasm			
	HDAC7	Nucleus/Cytoplasm			
	HDAC9	Nucleus/Cytoplasm			
IIB	HDAC6	Cytoplasm	Hda1	Zn^2+^	Specific
	HDAC10	Nucleus			
IV	HDAC11	Nucleus/Cytoplasm (depends on cell types and/or cellular environment)		Zn^2+^	Ubiquitous
SIRTUINS (Class III)	SIRT1	Nucleus	Sir2	NAD^+^	Variable
	SIRT2	Cytoplasm			
	SIRT3	Mitochondria			
	SIRT4	Mitochondria			
	SIRT5	Mitochondria			
	SIRT6	Nucleus			
	SIRT7	Nucleus			

#### The GNAT superfamily

GNAT, referred to as Gcn5-related-*N*-acetyltransferase, is the largest group among the KAT families. The members of the GNAT superfamily have conserved sequence motifs, especially the acetyl-CoA binding motif. The GNAT superfamily has several subfamilies. The HAT family is best studied in the midst of GNATs with GCN5 and PCAF as representatives. GCN5 was first identified in yeast as a transcriptional adaptor. Later, *Tetrahymena* GCN5 was reported as transcriptional-associated HAT type A^23^. Mammalian GCN5 and PCAF contain a HAT domain, a bromodomain, and PCAF-specific N-terminal domains^24^. For histone modifications, GCN5 shows enzymatic activity preference towards histone H3 at K9 and K14 and histone H4 at K8 and K16^25^. PCAF is one of the first acetyltransferases that was identified to have acetyltransferase activity towards non-histone proteins. A well-known substrate of PCAF is p53^26^. In addition to the GCN5/PCAF family, HAT1, Nut1, Elp3, Hpa2/Hpa3, MCM3AP, Eco1, and ARD1, belonging to the GNAT superfamily, exhibit acetyltransferase activity toward not only nuclear substrates but also cytosolic substrates. Because many ARD1 substrates have been investigated, the mechanistic regulation and biological functions of ARD1 will be discussed in detail in this review.

#### The MYST family

The acronym MYST was established on the basis of the first four members, namely: human **M**OZ, yeast **Y**bf2/Sas3, yeast **S**as2, and mammalian **T**IP60. The MYST family of acetyltransferases has been found in all eukaryotes. In humans, five members of MYST have been identified, which are as follows: (1) MOF (MYST1 or KAT8), (2) HBO1 (MYST2 or KAT7), (3) MOZ (MYST3 or KAT6), (4) MORF (MYST4 or KAT6B), and (5) TIP60 (KAT5)^24^. The MYST family of proteins contains an evolutionarily conserved MYST domain composed of an acetyl-CoA-binding site and a C2HC zinc finger, except for the absence of C2HC zinc finger in yeast Esa1. Aside from the MYST domain, individual proteins in this family carry additional domains, such as the chromodomain, N-terminal part of Enok, plant homeodomain (PHD) zinc finger, and serine/methionine-rich domain, which play a unique role in the functions of each protein (reviewed in^24^). The MYST family has diverse functions in not only chromatin dynamics and gene regulation by acting on histone but also cell homeostasis and metabolism regulation by acting on non-chromatin substrates^27^.

#### The p300/CBP (CREB-binding protein) family

Unlike the other two acetyltransferase families, which have homologs in different evolutionary organisms, the p300/CBP family comprises p300/CBP, which is metazoan specific, and Rtt109, which is yeast specific^28^. Interestingly, Rtt109 displays neither sequence conservation nor functional similarity to other acetyltransferases but has a structure homologous to the p300/CBP HAT domain^29^. p300 and CBP have very well-preserved regions: a catalytic acetyltransferase domain, a nuclear receptor-interacting domain at the N-terminus, a bromodomain, three cysteine/histidine-rich (C/H) domains with PHD in C/H2, a KIX domain, and an interferon-binding domain at the C-terminus^28^.

### Deacetylation

The balance of acetylation and deacetylation maintains cellular homeostasis. An imbalance of these modifications causes diseases and disorders. KDACs or so-called HDACs are different from KATs since this group of enzymes deacetylates not only acetylated histone proteins but also acetylated non-histone proteins. According to the homology of HDACs with yeast HDACs, 18 members of human HDACs are classified into two families. The conventional HDAC family, comprising 11 enzymes (HDAC1-11), is divided into four classes on the basis of their activity via a Zn^2+^-dependent mechanism, whereas the sirtuin family with seven enzymes (SIRT1-7) requires NAD^+^ as a cofactor for catalytic activity (Table 1)^30^.

#### Class I HDACs

Class I HDACs consist of HDAC1, HDAC2, HDAC3, and HDAC8, which are homologous to yeast Rpd3. This class of HDACs is predominantly localized to the nucleus and ubiquitously expressed in human cell lines and tissues^31^. HDAC1 and HDAC2 generally exist in multicomponent stable complexes, at least in three complexes—Sin3, NuRD, and CoREST—targeting different substrates. HDAC3 can form complexes with HDAC4, HDAC5, and HDAC7 in the presence of the coactivator SMRT or N-CoR^32^. The catalytic domain of HDAC8 has a nuclear localization signal, which is consistent with its main expression in the nucleus. HDAC8 can deacetylate its substrates without the presence of additional proteins^33^.

#### Class IIA HDACs

Class IIA is composed of four members: HDAC4, HDAC5, HDAC7, and HDAC9. Class IIA HDACs share sequence similarity to yeast Hda1. Similar to class I, class II HDACs share a homologous conserved catalytic domain. These proteins mainly act as transcriptional corepressors by deacetylating nucleosomal histones^34^. This class of deacetylases recruits class I proteins, particularly HDAC3, for repression activity. Class IIA HDACs show tissue-specific expression, indicating their differential roles in development.

#### Class IIB HDACs

HDAC6 and HDAC10 compose class IIB HDACs. HDAC6 and HDAC10 possess two deacetylase domains and a zinc finger domain at the C-terminus. HDAC6 mainly localizes to the cytoplasm and displays deacetylase activity towards cytoplasmic substrates, such as α-tubulin and cortactin^35^. The functions of HDAC10 are still unclear. Previous studies have implied that HDAC10 is predominantly localized to the nucleus in normal cells and to the cytoplasm in cancer cells^36^. HDAC10 potentially serves as a polyamine deacetylase^37^ and promotes cancer development, although its substrates are still unknown^38^.

#### Class IV HDAC

To date, HDAC11 is the only characterized enzyme of class IV HDAC. The subcellular localization of HDAC11 depends on cell types and/or the cellular environment. The expression of HDAC11 has been observed in the kidney, heart, brain, skeletal muscle, testis, and hematopoietic cells. HDAC11 has immune system-related and cell survival-related functions^39,40^. Similar to HDAC10, HDAC11 is poorly understood.

#### Sirtuin class

Class III HDACs include seven human sirtuins, which have two enzymatic activities: mono-ADP-ribosyltransferase and histone deactylase^41^. Sirtuins have sequence similarity to the yeast protein Sir2. SIRT1, SIRT2, and SIRT3 are considered to be true deacetylase enzymes. SIRT6 presents weak deacetylase activity and preferential ADP-ribosyltransferase activity. SIRT5 functions as a desuccinylase and demalonylase on carbamoyl phosphate synthetase and substrates other than deacetylase. SIRT7 specifically deacetylates histone H3 at lysine residue 18. Unlike other sirtuins, SIRT4 has ADP-ribosyltransferase and lipoamidase activities in addition to its deacetylase activity^42^. Sirtuins are expressed in various cell organelles. Each individual enzyme has a precise localization. SIRT1 is found primarily in the nucleus, but it shuttles to the cytoplasm under controlled conditions^43^. In contrast, SIRT2 is highly expressed in the cytosol, but it translocates to the nucleus during the G2/M transition^44^. SIRT6 and SIRT7 are nuclear proteins. In particular, SIRT3, SIRT4, and SIRT5 are expressed in the mitochondria, subsequently affecting metabolism regulation^45^.

## ARD1-mediated protein lysine acetylation

ARD1 was originally identified as a NAT in yeast, and it acetylates the N-terminal amino acid of newly synthesized proteins from the ribosome^12^. However, mammalian ARD1 has been revealed to have KAT and NAT activities^13^. In the last decade, various proteins have been shown to be acetylated by ARD1 at their internal lysine residues^46,47^. In this review, we summarize the diverse cellular functions regulated by ARD1-mediated lysine acetylation and describe how the KAT function of ARD1 is regulated in cells.

### KAT functions of ARD1

#### Cellular stress response

Heat shock protein (Hsp) 70 is a molecular chaperone that protects cellular proteins under stress conditions. It maintains cellular protein homeostasis by two opposing functions, protein repair and degradation^48^. Recently, Seo et al. found that in response to stress stimuli, such as reactive oxygen species (ROS), Hsp70 is rapidly acetylated at the K77 residue by ARD1 and then deacetylated again by HDAC4^49^. Depending on the acetylation/deacetylation status, Hsp70 switches its function between protein repair and degradation. This switch is essential for maintaining protein homeostasis and cell survival under stress conditions. Therefore, ARD1-mediated acetylation of Hsp70 is a key regulatory mechanism that protects cells against stress conditions. On the basis of its anti-apoptotic properties, Hsp70 has been recognized as a promising target for cancer therapy; however, the development of a selective Hsp70 inhibitor is not easy because of the high similarities between members of the Hsp70 protein family^50^. It is worth noting that the K77 residue is specific for Hsp70 and not conserved in other members of the Hsp70 family. Indeed, the K77R mutation, which inhibits the acetylation of Hsp70, increased the susceptibility of cancer cells to anti-cancer drugs^49^. In addition to cancer treatment, Hsp70 acetylation also has a significant effect on neuroprotection. The K77Q mutation, which mimics the acetylation state of Hsp70, inhibited neuronal cell death due to neurotoxins in vitro and in vivo^49^. These results suggest that targeting ARD1-mediated Hsp70 acetylation could be helpful in the treatment of various diseases, such as cancer, neurodegeneration, and inflammatory diseases, in which Hsp70 function is dysregulated.

In addition, oxidative stress has a dual role in cell growth and death; thus, there is a question of whether ARD1 could have opposing functions in ROS-mediated cell proliferation and cell death. Indeed, Shin et al. reported that ARD1 negatively regulated the oxidative stress response^51^. ARD1-overexpressing cells were more susceptible to oxidative stress than control cells, and ARD1 transgenic mice showed more severe injuries in the kidney and liver under hyperoxic conditions than wild-type mice. As a molecular mechanism, Shin et al. suggested that ARD1 acetylates the K49 residue of methionine sulfoxide reductase A (MSRA), which is a thioredoxin-linked enzyme that converts methionine sulfoxide to methionine to protect amino acids from oxidative stress, resulting in the reduction of enzymatic activity of MSRA^52^. ARD1 could possibly have opposing roles between cell survival and cell death in response to oxidative stress, probably depending on the intensity or period of oxidative stress. These opposing functions might be mediated by acetylating different substrate proteins. While ARD1 protected cells against ROS through Hsp70 acetylation^49^, ARD1 worsened ROS-induced cellular damage by MSRA acetylation^51^. Therefore, although ARD1 plays a critical role in cellular redox balance, when we target ARD1 in oxidative stress-relative diseases, the opposing functions of ARD1 should be considered, and the downstream substrate proteins acetylated by ARD1 should also be carefully reviewed in relevant diseases.

#### Autophagy

Autophagy is a cellular defense system in response to cellular stress and plays a crucial role in maintaining cell homeostasis^53^. Recently, Qian et al. found that the KAT activity of ARD1 is needed to initiate autophagy and promote cell survival under harsh conditions^54,55^. Under glutamine deprivation and hypoxic conditions, ARD1 acetylates phosphoglycerate kinase 1 (PGK1) at the K388 residue, and then, acetylated PGK1 subsequently phosphorylates the S30 residue of Beclin1. Beclin1 is a core protein of the protein complex that contains vacuolar sorting protein 34 (VPS34) and class III phosphatidylinositol-3 kinase (PI3K). Phosphorylated Beclin1 enhances the activity of this protein complex by increasing the binding of phosphatidylinositol to VPS34 and thereby increases phosphatidylinositol-3-phosphate (PtdIns3P) generation, which is a required molecular event for autophagosome formation^56^. Therefore, ARD1-mediated PGK1 acetylation is essential for glutamine deprivation- and hypoxia-induced autophagy. PGK1 acetylation by ARD1 promotes brain tumor formation, and indeed, PGK1 K388 acetylation levels are correlated with poor prognosis in glioblastoma patients^54^. This report reveals the important roles of ARD1 in the regulation of cell metabolism and suggests that the K388 acetylation level of PGK1 could be useful as a prognosis marker in cancer patients.

#### Cell cycle

Yeast ARD1, as its name indicates, has an important role in the life cycle. Thus, human ARD1 may have similar biological functions related to cell cycle regulation. Lim et al. first investigated the function of human ARD1 in lung cancer cells and showed that the depletion of ARD1 resulted in a significant defect in cell cycle progression, especially at the G1-S phase^57^. Regarding the molecular mechanism, Lim et al. suggested that ARD1 acetylates β-catenin; however, the acetylated lysine site of β-catenin has not been identified yet. Later, Seo et al. also showed that ARD1-overexpressing cancer cells enhanced tumor growth in a mouse xenograft model and that the eradication of KAT activity of ARD1 significantly prevented cell proliferation in vitro and in vivo^58^. Wang et al. found that ARD1 is upregulated in human prostate cancer patients and promotes prostate cell proliferation in vitro and in vivo^59,60^. Mechanically, ARD1 acetylates the K618 residue of androgen receptor (AR). The K618R mutation, which prevents the ARD-mediated acetylation of AR, attenuated the proliferation of prostate cancer cells in vitro and in vivo. The regulation of AR signaling by ARD1 is reviewed more exhaustively in another article written by Liu’s group in this issue. Recently, Lee et al. also found that ARD1 controls cell cycle progression by regulating cellular dNTP levels^61^. Cellular dNTPs are the building blocks of DNA. Because balanced cellular dNTP levels are critical for proper DNA replication and repair, cellular dNTP levels are strictly controlled by two types of enzymes, dNTP synthetase and dNTPase. SAM domain and HD domain containing protein 1 (SAMHD1) is the only known dNTPase that cleaves dNTPs^62^. Since SAMHD1 was first identified in immune cells, where SAMHD1 inhibits retroviruses, such as human immunodeficiency virus type 1 (HIV-1), by the depletion of cellular dNTPs to block retroviral replication, most studies have focused on the anti-viral activity of SAMHD1 in immune cells^63,64^. However, SAMHD1 is ubiquitously expressed in human organs, suggesting that it has additional biological functions in non-immune cells. Lee et al. first reported the oncogenic characteristic of SMAHD1 in cancer cells and showed that the dNTPase activity of SAMHD1 is stimulated by ARD1-mediated acetylation^61^. SAMHD1 is acetylated at the K405 residue by ARD1, and acetylated SAMHD1 exhibits enhanced dNTPase activity compared with non-acetylated SAMHD1. During the cell cycle, SAMHD1 acetylation is the highest in the G1 phase, and a mutation of SAMHD1 that inhibits its acetylation induces a defect in the G1/S phase transition and inhibits cellular growth. These results suggest that regulation of the cellular dNTP pool could be useful for inhibiting cancer development and that modulation of the KAT activity of ARD1 or SMAHD1 acetylation level is a possible approach for controlling the cellular dNTP pool.

#### Development

Since ARD1 was first identified to have important roles in yeast growth and sporulation, many studies on ARD1 focused on its functions related to cell growth or cell cycle. However, ARD1 is expressed in many types of cells; thus, ARD1 was believed to have additional biological functions in addition to the regulation of cell proliferation. Yoon et al. investigated the role of ARD1 during the developmental stage and found a novel function of ARD1 in bone development^65^. During osteogenesis, ARD1 expression was induced by Runt-related transcription factor 2 (Runx2), which led to osteoblast differentiation by the transactivation of osteoblast marker genes and negative control of Runx2 activity by ARD1. ARD1 acetylates Runx2 at the K225 residue, and this acetylation interferes with the binding of Runx2 to CBFβ, inhibiting the transcriptional activity of Runx2. ARD1 transgenic neonatal mice showed delayed bone development relative to their littermates, whereas many bones of ARD1 knockout mice were denser and more extended. The osteoblast differentiation of primary osteoblasts stimulated by BMP-2 was also negatively regulated by ARD1 in vitro. Moreover, the depletion of ARD1 augmented bone regeneration in a critical-size calvarial defect rat model, suggesting a new strategy for facilitating fracture healing by modulating ARD1 activity. In addition to bone development, ARD1 knockout mice displayed severe developmental defects, including partial embryonic lethality, growth retardation, brain disorders, and maternal effect lethality, although the relation between the KAT activity of ARD1 and these defects is unclear^66^. In humans, a Ser37Pro mutation in ARD1, which results in an ARD1 that lacks NAT activity, is known to cause genetic diseases, but the effect of this mutation on the KAT activity of ARD1 is also unknown^67^. The roles of ARD1 in development and genetic disease are addressed at some length in other articles written by Oh’s group and Lyon’s group in this issue.

#### Cell motility

On the basis of previous studies that reported that ARD1 is closely related to the cell cycle, Vo et al. traced the cellular location of ARD1 during the cell cycle and observed that ARD1 is colocalized with Aurora kinase A (AuA) in the centrosome during cell division^68^. AuA is a mitotic serine/threonine kinase that functions in centrosome maturation and separation and spindle assembly during mitosis^69^. Vo et al. showed that ARD1 directly acetylates the K75 and K125 residues of AuA and that this event is essential for the kinase activity of AuA^68^. Double mutations at K75R/K125R decreased the kinase activity of AuA and caused severe defects in cell migration. Since AuA is known to be overexpressed in many cancers and is correlated with metastasis and poor prognosis, Vo et al. suggested that ARD1-mediated AuA probably contributes to cancer development. In contrast to this suggestion, Shin et al. proposed that ARD1 downregulates tumor cell migration and invasion by myosin light chain kinase (MLCK) acetylation. MLCK is a calcium/calmodulin-dependent serine/threonine kinase that phosphorylates MLC and is implicated in a variety of cellular functions, including cell contraction and migration^70^. Shin et al. suggested that ARD1 acetylates the K608 residue of MLCK in vitro and in vivo, reducing the kinase activity of MLCK. Even though the effect of ARD1 on cell motility is not clear, since AuA and MLCK are expressed in diverse types of human cells, such as endothelial cells, neuronal cells, and smooth muscle cells, it is possible that ARD1 regulates diverse biological events related to cell motility, such as angiogenesis, vascular permeability, neurite extension, transmitter release on synapse formation, and muscle contraction.

### Regulation of the KAT activity of ARD1

Many kinases and acetyltransferases catalyze themselves to stimulate enzymatic activities in a process called autophosphorylation or autoacetylation^71,72^. Seo et al. found that ARD1 acetylates itself at the K136 residue, and this modification is essential for the functional activation of ARD1 as a KAT^58^. The K136R mutation, which inhibits the autoacetylation of ARD1, abolished the KAT activity of ARD1. A number of studies have linked ARD1 to cancers, and human ARD1 has been increasingly considered to be a therapeutic target for cancer treatment^47^. Notably, the inhibition of human ARD1 autoacetylation by the K136R mutation blocked cancer growth in vitro and in vivo by the downregulation of cyclin D1, suggesting modulation of the autoacetylation of ARD1 for cancer therapy^58^. Autoacetylation is a key switch for regulating the KAT activity of ARD1; however, the question still remains whether the autoacetylation of K136 also affects the NAT activity of ARD1. If this modification regulates only the KAT activity and not the NAT activity of ARD1, comparison studies of the cellular functions of K136R mutant ARD1 and the dominant negative mutant (DN), which lacks both NAT and KAT activities, could offer clues on how the KAT and NAT functions of ARD1 are differentially regulated in cells.

**Fig. 2:**
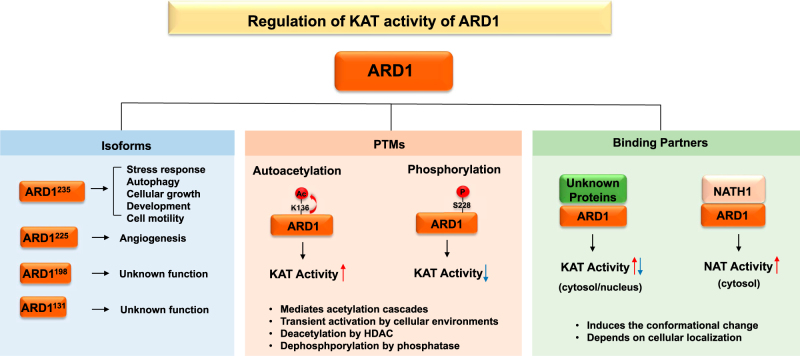
Regulation of the KAT activity of ARD1. The KAT activity of ARD1 is regulated by PTMs. While autoacetylation at the K136 residue stimulates the KAT activity of ARD1, phosphorylation at the S228 residue inhibits the KAT activity of ARD1. Autoacetylation is conserved among ARD1 isoforms, including ARD1^235^ and ARD1^225^; however, autoacetylation regulates differential cellular functions depending on the isoform. While the autoacetylation of ARD1^235^ enhances cellular growth, the autoacetylation of ARD1^225^ inhibits angiogenesis. The KAT activity of ARD1 might also be regulated by unknown binding proteins

The intriguing part is that autoacetylation is conserved among diverse ARD1 variants derived from alternative RNA splicing^73^; however, it regulates differentially the roles of ARD1 variants in tumorigenesis. The characteristics and biological function of ARD1 variants are reviewed in other articles in this issue written by Chung’s group. Seo et al. found that among several ARD1 variants, human ARD1^235^, mouse ARD1^235^, and mouse ARD1^225^ recombinants undergo similar autoacetylation with the target site conserved at K136 in vitro^74^. While autoacetylation of human ARD1^235^ and mouse ARD1^235^ contributed to cancer growth in normoxic conditions, mouse ARD1^225^ autoacetylation had no effect on cellular growth. Instead, autoacetylation of mouse ARD1^225^ prevented tumor angiogenesis by the degradation of hypoxia-inducible factor-1α (HIF-1α) protein under hypoxic conditions. Autoacetylation stimulated the catalytic activity of mouse ARD1^225^ to acetylate the K532 residue of HIF-1α, leading to the proteasomal degradation of HIF-1α. The autoacetylation of ARD1 variants selectively regulates cancer growth and angiogenesis in an isoform-specific manner (Fig. 2).

Whereas autoacetylation stimulates the KAT activity of ARD1, phosphorylation inhibits the KAT activity of ARD1. Qian et al. found that mTOR directly phosphorylates the S228 residue of ARD1 in vitro, and this phosphorylation inhibits the KAT activity of ARD1, which is especially required for PGK1 acetylation^54^. Under glutamine deprivation or hypoxic conditions, mTOR-induced ARD1 phosphorylation was downregulated, and ARD1 was activated and consequently acetylated PGK1, leading to autophagy promotion. Therefore, the KAT activity of ARD1 may be balanced by various post-translational modifications, including acetylation and phosphorylation, which are stimulated by certain cellular stimuli (Fig. 2). Further studies are needed to elucidate how these modifications coordinate with each other to regulate the KAT activity of ARD1.

Regarding the KAT activity of ARD1, we should not overlook the fact that the KAT activity of ARD1 requires delicate handling; it seems to be influenced by various experimental conditions. Indeed, previous reports presented conflicting data about the KAT activity of ARD1. Murray-Rust et al. observed that recombinant ARD1 underwent self-mediated N-terminal acetylation in vitro, suggesting that ARD1 has NAT activity rather than KAT activity^75^. Magin et al. also argued for the KAT activity of ARD1 because they observed no difference in the lysine acetylation of substrate proteins, such as MSRA, MLCK, and RUNX2, with or without recombinant ARD1 in vitro, suggesting that the substrates may be acetylated chemically^76^. However, Sanchez-Puig et al. characterized the fibrillar conformation of recombinant ARD1^77^. Size exclusion chromatography and electron micrograph revealed the presence of soluble aggregates of recombinant ARD1. Consistent with this finding, Seo et al. found that the KAT activity of recombinant ARD1 easily disappeared and that only freshly prepared recombinant ARD1 showed KAT activity, suggesting that recombinant ARD1 formed aggregates over time^58^. Therefore, the labile catalytic activity of recombinant ARD1 could be one explanation for the conflicting results on the KAT activity of recombinant ARD1. Since autoacetylation is essential step to stimulate the KAT activity of ARD1, the presence of KAT activity could be determined by the autoacetylation status of recombinant ARD1.

In cells, the basal level of KAT activity of ARD1 might be not sufficient for detection because the KAT activity of ARD1 was upregulated briefly by a specific cellular stimulus. Qian et al. found that the KAT activity of ARD1 required to acetylate the lysine residue of PGK1 was usually inhibited under normal conditions and that this activity was stimulated only after glutamine deprivation or hypoxia stimulation^54^. Similarly, Seo et al. showed that the autoacetylation of ARD1 rapidly increased with hydrogen peroxide or 1-methyl-4-phenylpyridinium (MPP^+^) treatment and that it disappeared soon after^49^. Therefore, the KAT activity of ARD1 seems to be transiently upregulated through autoacetylation in response to certain cellular stimuli. Furthermore, the autoacetylation of ARD1 stimulated Hsp70 acetylation, which was followed by the HDAC4-mediated deacetylation of Hsp70^49^. These results propose the existence of an “acetylation cascade” in which proteins are sequentially activated by acetylation to amplify the original signal and cross-talk with different signals, similar to the phosphorylation cascade (Fig. 2). Like the phosphorylation and dephosphorylation induced by kinases and phosphatases, acetylation cascades could be regulated by reversible acetylation and deacetylation reactions mediated by KATs and HDACs, respectively. As most PTMs are transient, ARD1-mediated-protein acetylation is probably maintained briefly. Therefore, to detect the lysine acetylation of ARD1 substrate protein or discover the acetylation cascades regulated by ARD1 in cells, the specific physiological conditions that transiently stimulate the KAT activity or autoacetylation of ARD1 should be determined first. The labile and transient characteristics of the KAT activity of ARD1 in vitro and in vivo should be treated with caution when investigating the biological functions of ARD1 as a KAT.

As the NAT function of ARD1 is regulated by binding with NATH, the KAT functions of ARD1 could be regulated by binding with diverse proteins (Fig. 2). Unlike its NAT activity, the KAT activity of ARD1 does not seem to need NATH binding since recombinant ARD1 acetylates the internal lysine residues of its substrate proteins in vitro without NATH. In addition, NATH protein is located in only the cytosol, whereas ARD1 is localized in both the nucleus and cytosol, suggesting independent roles of ARD1 that do not involve NATH. Beyond NATH, little is known about the binding proteins of ARD1 so far; thus, the identification of unknown binding partners of ARD1 that regulate KAT activity might be challenging. For example, proteome analysis under various cellular conditions that stimulate the KAT activity of ARD1 could be helpful for identifying critical molecules for the KAT functions of ARD1.

## ARD1 and human diseases

The expression of ARD1 is universal across cells. Furthermore, ARD1 displays distinct activity dependent on its sole activation or its activation as a subunit of NatA or its subcellular localization. Numerous substrates of ARD1 have been identified. Hence, ARD1 is a critical player in pathological progression.

### Cancer

Many studies have demonstrated the association between the overexpression of ARD1 and cancer progression and poor prognosis. A previous study on ARD1 expression in 19 common types of cancers in human tissues revealed that ARD1 is highly expressed in cancer tissues: adenocarcinoma, squamous cancer and especially breast cancer, bladder cancer, stomach carcinoma, cervical carcinoma, hypothyroid carcinoma, lung cancer, and intestinal cancer. Furthermore, there is a significant difference in ARD1 expression between cancer tissues and adjacent tissues in the same tissue section^78^. ARD1 has also been found to be overexpressed in colorectal cancer^79^, hepatocellular carcinoma^80^, lung cancer^81^, and prostate cancer^59^. In a previous study, targeting of ARD1 by microRNA repressed the tumorigenesis of colon cancer in vitro, suggesting that ARD1 acts as an oncogene in colon cancer^82^. The KAT activity of ARD1 is probably the mechanism underlying the contribution of ARD1 to cancer progression. Indeed, ARD1 has been reported to promote cell proliferation by regulating cyclin D1. The first study that reported the link between ARD1 and cyclin D1 used lung cancer cells. This study showed that ARD1 acetylated β-catenin and activated the binding of β-catenin/TCF4 to the cyclin D1 promoter. The depletion of ARD1 by siRNA in H1299 and A549 cells caused the transcriptional repression of cyclin D1, leading to the arrest of G1 and inhibition of cell proliferation^57^. ARD1 also exhibits autoacetylation at the K136 residue, resulting in the activation of AP-1, which is a transcriptional regulator of cyclin D1 during tumorigenesis, thereby increasing cyclin D1 expression. Consequently, A549 cell proliferation is promoted and tumorigenesis is enhanced in a xenograft model with H460 lung cancer cells^58^. Similarly, the overexpression of ARD1 in lung cancer cells showed the positive regulation of ARD1 in DNMT1 enzymatic activity, mediating E-cadherin silencing and thus inducing cell proliferation and xenograft tumor formation^81^. The androgen receptor (AR) has been reported to be a substrate of ARD1. AR acetylation by ARD1 generates AR-mediated gene transcription involved in tumor formation, promoting prostate tumorigenesis^59^. In addition, a previous study showed that the acetylation of SAMHD1 by ARD1 intensifies the dNTPase activity of SAMHD1, which promotes cell cycle progression in cancer cells and increases the expression level of cyclin D1 and cyclin B1 and promotes cell proliferation^61^. In addition, ARD1 also participates in governing cell cycle progression by regulating AuA acetylation. AuA, which overrides mitosis in cancer cells, is acetylated by ARD1; its activity is enhanced, and the G2/M transition is accelerated, contributing to cell proliferation. Moreover, the acetylation of AuA stimulates cell mobility, which is a crucial trait of metastasis in cancer development, by activating the p38/AKT pathway^68^.

ARD1 also contributes to tumorigenesis by inhibiting cell death. Silencing of ARD1 causes the cell to become susceptible to daunorubicin, triggering apoptosis^83^. Park et al. found that the interaction of ARD1 and RIP1 via the acetyltransferase domain is involved in doxorubicin-induced NF-κB activation, preventing cell death^84^. In addition, Xu et al. elucidated that the depletion of ARD1 sensitizes cancer cells to stress-inducing agents, including oxidative stress-inducer H_2_O_2_, mitotic stress-inducer Taxol, and DNA-damage inducer 5-fluorouracil, because of the requirement of ARD1 to interact with RelA/p56 and activate MCL1 transcription for the prevention of apoptosis^85^. Another function of ARD1 is protecting cells from death via the acetylation of Hsp70. The acetylation of Hsp70 by ARD1 stimulates the formation of Hsp70/Apaf-1 and Hsp70/AIF complexes, consequently preventing caspase-dependent and caspase-independent apoptosis, respectively. ARD1-mediated Hsp70 acetylation also attenuates autophagic cell death, which is linked to programmed cell death^86^. However, autophagy plays binary roles in cancer. On one hand, autophagy functions as a pro-death mechanism under stress conditions, as it can trigger apoptosis via several proteins with dual roles in autophagy and apoptosis, such as Beclin1, Bcl-2, Atg5, and p53^87,88^. On the other hand, autophagy can enable tumor cell survival under metabolic stress^88,89^. Recently, ARD1 was found to regulate PGK1-phosphorylated Beclin1 by acetylating PGK1, which in turn stimulates autophagy activation under glutamine deprivation and promotes brain tumorigenesis^54^. ARD1 has been demonstrated as a tumor suppressor in breast cancer, and the occurrence of ARD1 is correlated with a better clinical outcome^90,91^; this finding is possibly associated with the NAT activity of ARD1 rather than its KAT activity^90^. Moreover, ARD1 is required for caspase activation under stress conditions^92^. Thus, ARD1 acts as an oncogene or tumor suppressor because of its localization, stress conditions, and tissue specificity.

### Neuronal diseases

Many studies on the functions of ARD1 in neuronal regulation have been performed. The overexpression of mutant ARD1 and silencing of ARD1 limit the dendritic development of cerebellar Purkinje cells, which means that ARD1 is crucial for neuronal dendritic development^93^. Amyloid β-protein (Aβ) is a product of amyloid precursor protein (APP) proteolysis, and the accumulation of Aβ in the brain is a well-known cause of Alzheimer’s disease (AD)^94^. ARD1 in a complex with NAT1 has been investigated to suppress APP endocytosis correlated with the inhibition of Aβ secretion, suggesting the protective role of ARD1 in AD^95^. However, the mechanism underlying Aβ suppression is still unclear. Further studies are required to clarify how the ARD1/NAT1 complex suppresses Aβ production. Huntington’s disease (HD) is another progressive neurodegenerative disorder. A hallmark of HD is the aggregation of huntingtin (HTT) and formation of cytoplasmic and nuclear inclusions in the brain^96^. Arnesen et al. proposed that ARD1 in a complex with NatA interacts with HTT interacting protein (HYPK), which displays chaperon-like activity at the ribosome, and prevents HTT aggregation. Knockdown of either HYPK or ARD1 increases the aggregation of HTT^97^. Thus, ARD1 as a subunit of NatA could possibly contribute to preventing the progression of HD, although the underlying mechanism has not yet been verified.

### Genetic diseases

The link between genetic diseases and protein acetylation was unknown until Rope et al. showed the association of an X-linked disorder with NAT in 2011. Lethality in male infants with severe global developmental delays, craniofacial anomalies, hypotonia, and cardiac arrhythmia is a distinct feature of this X-linked syndrome, which is termed Ogden syndrome. The cause could be the mutation c.109 T > C in *ARD1*, accounting for p.Ser37Pro, which results in a reduction of ARD1/NatA enzymatic activity^67^. This assumption was confirmed using a *Saccharomyces cerevisiae* model^20^. Later, it was revealed that the ARD1 S37P mutant shortens helix α2 and changes ARD1 flexibility, negatively affecting NatA complex activity and thereby impairing the acetylation of ARD1/NatA substrates, including THOC7^98^. Recently, Ogden syndrome was found in a female patient^99^.

In addition, Lenz microphthalmia syndrome, characterized by microphthalmia or anophthalmia, developmental delay, intellectual disability, skeletal abnormalities, and malformations of the teeth, fingers, and toes, is attributed to ARD1 mutation-induced retinoic acid pathway dysregulation. The splice donor mutation c.471 + 2 T > A generates ARD1 aggregation in the cytoplasm and the downregulation of retinol uptake^100^.

ARD1 also plays a role in different developmental delay phenotypes because of its mutated location. The mutations at c.319 G > T (p.Val107Phe) and c.346 C > T (p.Arg116Trp) in ARD1 cause severe non-syndromic developmental delay^101^. The missense variant c.128 A > C (p.Tyr43Ser) of ARD1 at the X chromosome induces developmental delay with long QT in patients^102^. A clinical study has shown that X-link recessive mutations p.Arg83Cys and p.Phe128Leu of ARD1 are correlated with ARD1 acetyltransferase activity^103^. It has been noted that a reduction in NAT activity in ARD1 mutants seems to produce more symptoms during developmental delay^101^.

## Conclusion

In the past 50 years, our understanding of protein acetylation has increased greatly since the study by Phillips. It is now widely known that protein acetylation plays a pivotal role in regulating gene transcription by modulating histone acetylation and physiological and metabolic processes by mediating the acetylation of either chromatin or non-chromatin proteins in various cell compartments from the nucleus to the membrane. ARD1, as a member of the KAT proteins, displays its vital roles in cellular regulation via its enzymatic activity towards its substrates. The precise subcellular localization of ARD1 is also a considerable issue. ARD1 and activation protein acetylation are administered by multiple delicate modifications. Overexpression, dysregulation or depletion of ARD1 functionally impacts cellular homeostasis, which is the cause of oncogenesis and neurodegeneration. Further studies are required to understand by which catalytic mechanism ARD1 acetylates its partners and how the conformation of ARD1 changes when it is regulated by other modifications, such as autoacetylation and phosphorylation, since the structure of human ARD1 has not yet been validated. However, the crystal structures of its homologs in lower evolutional species, such as *Thermoplasma volcanium*^104^, *Schizosaccharomyces pombe*^105^, *Sulfolobus solfataricus*^106^, and *Chaetomium thermophilum*^107^, have been investigated. The implication of ARD1 in tumorigenesis has been progressively studied, showing that ARD1 is a promising target for anti-tumor therapeutic approaches. The development of an ARD1 inhibitor is still required. Achievements in generating chemicals that target ARD1 would provide information on ARD1 and ARD1-related disease treatment.
